# Supplementary material for: Synergy between Cyclase-associated protein and Cofilin accelerates actin filament depolymerization by two orders of magnitude
Source: Nat Commun. 2019 Nov 22;10:5319. doi: 10.1038/s41467-019-13268-1 (PMC6876572; doi:10.1038/s41467-019-13268-1)
Supplement: Supplementary file 4 — Supplementary Information [file 41467_2019_13268_MOESM4_ESM.pdf]

Supplementary Materials for

Synergy between Cyclase-associated protein and Cofilin accelerates actin filament depolymerization by two orders of magnitude

S. Shekhar, J. Chung, J. Kondev, J. Gelles and B. L. Goode

Correspondence to: gelles@brandeis.edu; goode@brandeis.edu

Supplementary Fig. 1: Srv2/CAP and Cof1 synergistic depolymerization is observed across a wide pH range.

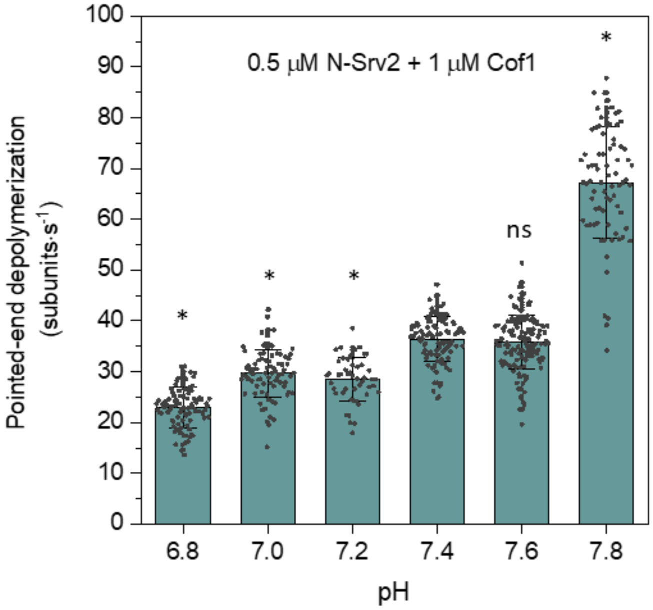

Rates (± sd) of pointed end depolymerization by 1 μM Cof1 and 0.5 μM N-Srv2 as a function of pH of the TIRF buffer. Statistical comparison with pH 7.4 condition: ∗, ($p < 0.05$) and ns, no evidence for significance at $p = 0.05$. Number of filament ends analyzed for each condition (left to right): 95, 84, 48, 107, 151, 76. All experiments were performed at least three independent times, and yielded similar results. Data shown are from one experiment.

Supplementary Fig. 2: Effects of varying the Cof1 concentration on synergistic depolymerization with Srv2/CAP.

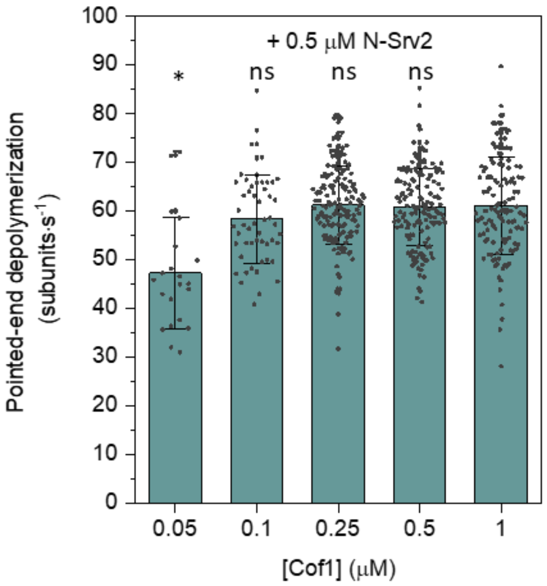

Rates (± sd) of pointed-end depolymerization at a fixed concentration of N-Srv2 (0.5 μM) and variable concentrations of Cof1. Statistical differences: ✳ ($p < 0.05$), ns (no evidence for significance at $p = 0.05$) compared with the 1 μM Cof1 + 0.5 μM N-Srv2 condition. Number of filament ends analyzed for each condition (left to right): 22, 52, 151, 150, 124. All experiments were performed at least three independent times, and yielded similar results. Data shown are from one experiment.

Supplementary Fig. 3: In presence of 20 mM P_i, increasing Cof1 concentration to 5 µM is sufficient to fully decorate actin filaments.

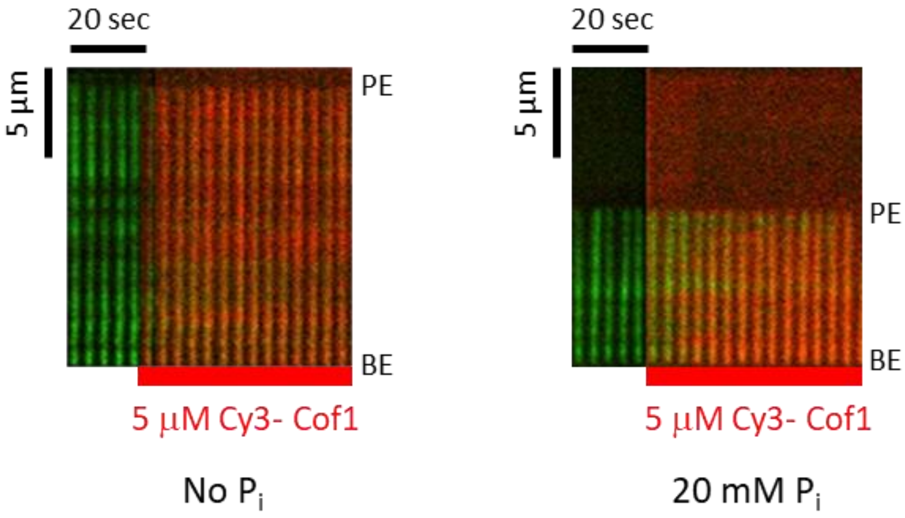

Merged two-color kymograph of an Alexa-488 labelled actin filament (green) being exposed to 5 µM Cy3-Cof1 (red) introduced at the beginning of the red bar in absence (left) and presence of 20 mM P_i (right). BE, barbed end. PE, pointed end. These experiments were performed two independent times, and yielded similar results. Images shown are from one experiment.

Supplementary Fig. 4: Fluorescently labeled Srv2ΔCARP exhibits a similar ability to synergize with Cof1 in depolymerization as unlabeled N-Srv2.

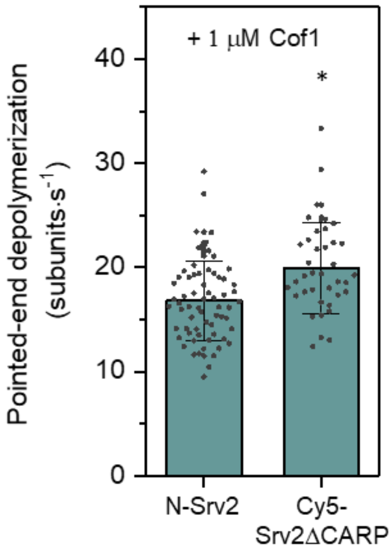

Rates (± sd) of pointed-end depolymerization for 42 nM N-Srv2 or Cy5-Srv2ΔCARP in the presence of 1 μM Cof1. Statistical differences: ✳ (p < 0.05) compared with the 42 nM N-Srv2 and 1 μM Cof1 condition. Number of filament ends analyzed for each condition (left to right): 68, 38. All experiments were performed at least three independent times, and yielded similar results. Data shown are from one experiment.

Supplementary Fig. 5: Step-photobleaching analysis of Cy5-Srv2ΔCARP molecules.

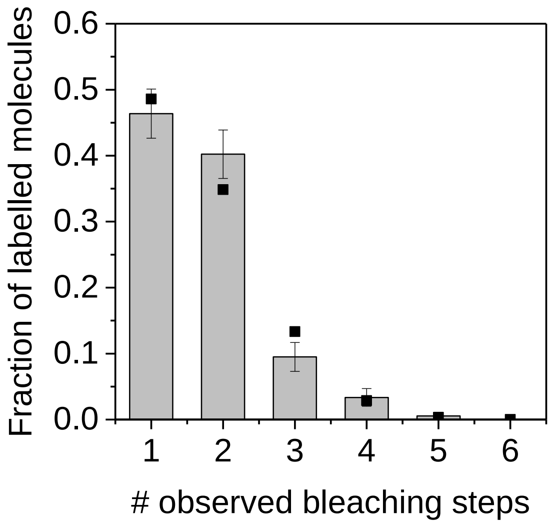

Fractions (± se) of surface-immobilized molecules with the indicated number of bleaching steps measured for *n* = 179 Cy5-Srv2ΔCARP molecules (grey), along with the binomial distribution predicted for hexamers (black) yielding a stoichiometry of 0.22 ± 0.01 Cy5 per Srv2ΔCARP monomer, consistent with the value measured colorimetrically (0.25). This experiment was performed two independent times, and yielded similar results. Data shown are from one experiment.

Supplementary Fig. 6: Cy5-Srv2ΔCARP molecules preferentially bind to the pointed ends rather than sides of Cof1-decorated actin filaments.

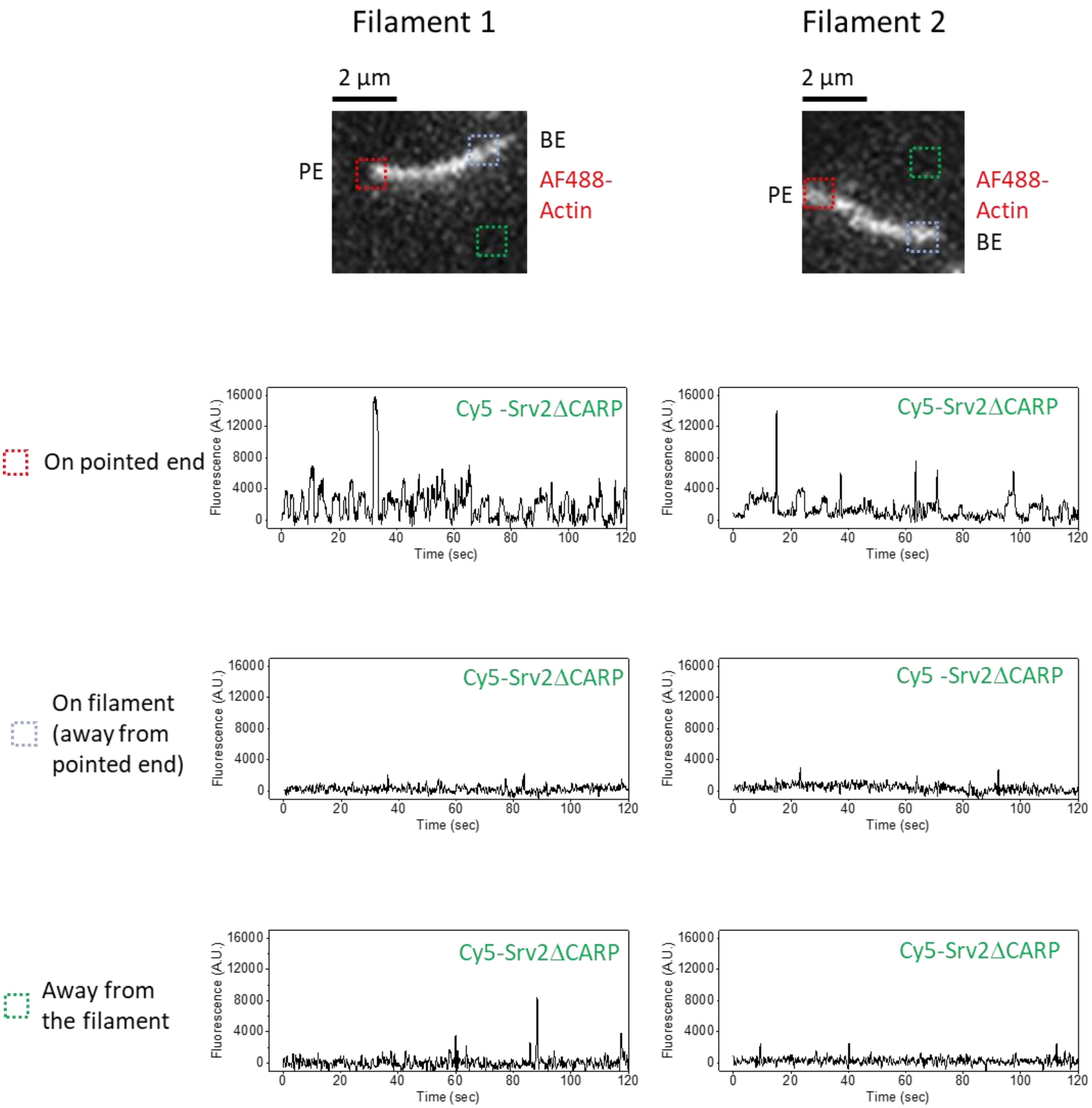

Shown are example recordings from two actin filaments at 1 µM Cof1 and 83 nM Cy5-Srv2ΔCARP. Top: Still image of Alexa-488 actin fluorescence with the pointed end (PE) and the

barbed end (BE) marked. Bottom: Time records of integrated Cy5-Srv2ΔCARP intensity from 5 x 5 pixel squares tracking the pointed end or centered at the on filament and away from the filament regions designated in the top images (colored boxes). The three regions correspond to the pointed end, a control region away from the filament, and a selected region on the filament away from the pointed end. Time resolution 0.065 s per frame, smoothed in a 0.71 s sliding window (2nd order polynomial, Savitzky-Golay filter). These experiments were performed two independent times, and yielded similar results. Images shown are from one experiment.

Supplementary Fig. 7: Intensity distributions of pointed end-associated Cy5-Srv2ΔCARP molecules.

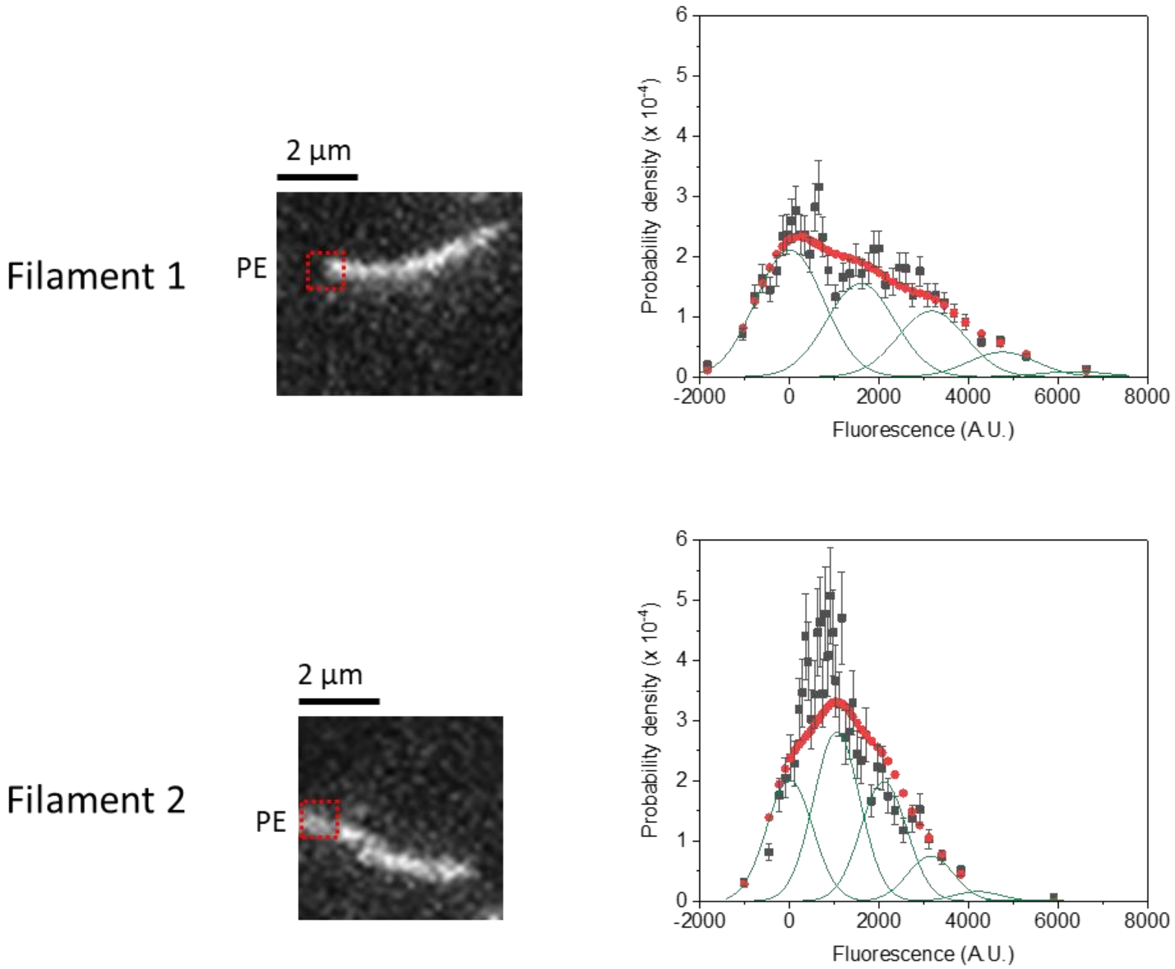

Background-corrected intensity distributions of pointed end-associated Cy5-Srv2ΔCARP fluorescent spots agree with that predicted from the step photobleaching of surface-immobilized individual Cy5-Srv2ΔCARP molecules. Probability density histograms (± se) integrated Cy5-Srv2ΔCARP intensity time points from 5 x 5 pixel squares centered at the pointed ends of the two filaments shown in Supplementary Fig. 6 (n = 1959 and 1459, respectively). Outlier data with intensity > 8,000 units (2% and 0.9%, respectively) were removed. Histogram was fit with seven equally spaced gaussian peaks representing the pointed end without fluorescence and the

pointed end with 1, 2, 3, 4, 5 or 6 Cy5 dyes on the end-bound Srv2ΔCARP hexamer. Relative heights of the peaks representing 1-6 dye molecules were fixed at the values determined in Figure 4B. The fit yielded Gaussian peak spacing, Gaussian width, and additional fractional amplitude of the no fluorescence peak (with 90% C.I.s) as 0.29 [0.23, 0.33], 757 [713, 802], and 1.59×10^3 [1.53, 1.64] for filament 1 and 0.05 [0.01,0.09], 516 [480,558] and 1.05×10^3 [1.01, 1.10] for filament 2. These experiments were performed two independent times, and yielded similar results. Data shown are from one experiment.
