# Supplementary material for: Synergy between Cyclase-associated protein and Cofilin accelerates actin filament depolymerization by two orders of magnitude
Source: Nat Commun. 2019 Nov 22;10:5319. doi: 10.1038/s41467-019-13268-1 (PMC6876572; doi:10.1038/s41467-019-13268-1)
Supplement: Supplementary file 7 — Description of Additional Supplementary Files [file 41467_2019_13268_MOESM7_ESM.pdf]

Title: Supplementary Movie 1.

Description: Cof1 and Srv2 synergize to accelerate actin filament depolymerization.

Merged two-color time-lapse movie of Alexa-488 labelled actin filaments (green) depolymerizing in the following conditions: Left - 1 μM Cy3-Cof1 (red) alone or Right - 1μM Cy3-Cof1 (red) & unlabeled 0.5 μM Srv2. Pointed ends of the two filaments are indicated with white asterisks. Please see Fig. 1d for kymographs of these two filaments.

Title: Supplementary Movie 2.

Description: Direct visualization of transient binding of Cy5-Srv2ΔCARP at the depolymerizing pointed end of a Cofilin-decorated actin filament.

Merged two-color time-lapse movie of Alexa-488 labelled actin filament (red) depolymerizing in the presence of 1μM Cof1 and 83 nM Cy5-Srv2ΔCARP (green). Filament pointed end is indicated by the white asterisk. Kymograph of depolymerization and Cy5-Srv2ΔCARP intensity measured at the pointed end are shown for this filament in Figs. 4c and 4d (left), respectively.

Title: Supplementary Movie 3.

Description: Quantitation of Cy5-Srv2ΔCARP fluorescence intensity at the pointed end of a depolymerizing, Cofilin-decorated actin filament.

Left: Time-lapse fluorescence images of an alexa-488 labelled actin filament depolymerizing in the presence of 1 μM Cof1 and 83 nM Cy5-Srv2ΔCARP. Magenta colored circle indicates the location of the pointed-end of the filament as detected from the binary image (center). Right: Fluorescence image in the Cy5-Srv2ΔCARP channel. The magenta square is centered at the location of the filament pointed-end detected from the binary image of the actin filament. This is the same filament as shown in Supplementary Movie 2 and Figs. 4c and 4d (left). Scale bar, 1 μm.
